# Assessing the Relationship Between Internet Banking and Investment Decision Through Sustainability and Competitive Advantage: Evidence From Congolese Banks

**DOI:** 10.3389/fpsyg.2022.869646

**Published:** 2022-06-03

**Authors:** Mengyun Wu, Jean Baptiste Bernard Pea-Assounga

**Affiliations:** School of Finance and Economics, Jiangsu University, Zhenjiang, China

**Keywords:** bank competitive advantage, bank investment decision, bank sustainability, internet banking, structural equation modeling, Republic of Congo

## Abstract

Competitive advantage and sustainability emerge as important factors for the success of an organization’s overall differentiation. This research aims to identify the relationship between internet banking and bank investment decision, as well as gaging the mediating effects of sustainability and competitive advantage as attributes of investment decisions. To achieve that, a questionnaire was administrated to banks’ employees and customers. To carry out the hypothesis testing, we have employed structural equation modeling through SPSS and SmartPLS. The findings suggest that internet banking, sustainability, and competitive advantage constructs are significant antecedents of banks because they highlight valuable attributes for banks to attain future benefits. This paper contributes to bank managers and scholars by providing a framework and supporting theories that help to identify relevant constructs and strategic resource characteristics. From the findings, we recommend conducting future studies in other countries or fields to generalize our results.

## Introduction

Recent advancements in IT have contributed to the rapid growth of modern and creative financial services known as financial technology (Fintech). Investment decision is a growing challenge in many business enterprises in the world. Investors are focusing on new ways to invest in their business for better performance; however, it seems to face difficulty in making investment decisions. According to Musau (2016) investment choices in terms of capital expansion decisions, renewal decisions, study, and replacement decisions are defined by financial management.

The investment decisions indicate “capital budgeting” in the language of financial management. The “investment decision-making and capital budgeting” in the finance industry are not deemed distinct acts. The concept “capital” is specifically perceived in the investment decision to relate to real assets that can take any form of construction, plants, equipment, raw materials, etc., while investment relates to any actual assets of this type. In other words, investment decisions must relate to issues of if today’s contributions to financial assets will rise tomorrow’s sales to cover expenses (Pea-Assounga and Wu, 2022). Investment decisions are thus, a pledge to cash capital at various periods in anticipation of potential economic returns. Among the various available resources for investment, a decision must be taken. Since these, investment decisions concern the option of purchasing real assets in a productive process over a period of time.

In nowadays era of globalization and the competitive marketplace, firms experience spectacular changes (Acar and Özşahin, 2018; Anwar, 2018; Pea-Assounga and Yao, 2021). Therefore, organizations have begun to look for sustainable business survival techniques and creative tactics (Anning-Dorson, 2018). In such circumstances, innovation technology refers here by internet banking as a key driver for competition, superior performance and leads to investment decisions (Evans et al., 2017; Latan et al., 2020). Fintech is a newly coined word for innovation in financial institutions and scholars have clarified it with various definitions and dimensions (Thakor, 2020). The key focus of innovation, however, relates to the competitive advantage of the organization and superior efficiency (Evans et al., 2017; Anwar, 2018). For example, Teece (2018) has recently identified innovation as an architecture that shows how an organization can build and produce consumer values. It relates directly to how the business operates and how shareholders’ values are created (Anwar, 2018). Besides that, it must be noticed that internet banking, apart from product and innovation services, relates to a company’s various disruptive practices, such as operation, structure, distribution, and engagement, which are carried out to achieve sustainability, superior efficiency, and competitive advantage. Investment decisions are the area in which recent scholars have gained sufficient attention (Warsame and Ireri, 2016; Božić and Botrić, 2017; Shahwan, 2018; Ogunlusi and Obademi, 2019; Steinmeier and Stich, 2019).

Innovation and investment decisions have been discussed in several top management journals. However, empirical research on the outcomes of innovation and investment decisions is still rare (Anning-Dorson, 2018). Nevertheless, innovation undeniably plays an important role in achieving sustainability, superior financial performance, competitive advantage, and investment decisions in dynamic markets (Evans et al., 2017; Anwar, 2018). This investigation discusses the concept of internet banking as an innovation component toward sustainability, competitive advantage, and investment decisions of banks operating in the developing marketplace of the Republic of Congo. The current study also aims to propose sufficient evidence and supports Decision theory (DT), Theory of Planned Behavior (TPB), and Resource-based View Theory (RBVT). For example, it is scrutinized that internet banking contributes considerably to the competitive advantage of companies operating in developing nations (Anning-Dorson, 2018). Conversely, researchers have overlooked the mediating effects of sustainability and competitive advantage, and it is uncommon to find an evidence analysis of this kind in the banking sector. This research will answer the following questions: “Does internet banking influence bank sustainability, bank competitive advantage, and banks’ investment decisions? Do sustainability and competitive advantage mediate the relationship?” The results are beneficial for managers and owners of banks who explore superior performance, sustainability, competitive advantage, and investment decisions.

We note that past works pointed to analyzing the impact of investment and financial decisions on a collection of the response variables, like financial performance, by reviewing relevant literature (Božić and Botrić, 2017; Weber, 2017; Balushi et al., 2018; YuSheng and Ibrahim, 2020). In comparison, the present investigation seeks to examine the impact of internet banking as a component of financial innovation on investment decisions in the presence of mediation constructs, such as bank sustainability and bank competitive advantage. In addition, previous research has been employed in foreign contexts (Carboni and Medda, 2019; Ogunlusi and Obademi, 2019; Barbu et al., 2021; Chen et al., 2021) whilst the present research has been applicable to the Congolese market, especially in the banking industry, one of the major financial sectors. Also, we relied on our research on Congolese banks, where financial innovations impact management, organization, market, and development. In addition, the ICT sector is anticipated to contribute to 4% of GDP in the Republic of Congo (World Bank, 2018; Pea-Assounga and Yao, 2021). As infrastructure improves, it is projected to contribute considerably more, especially in banking institutions and services, where it already plays a major role. Internet banking has given traditional financial services a fresh lease of life in the Republic of Congo. According to the World Bank (2018), banks in the Republic of Congo are experimenting with new technologies such as online banking and mobile banking, which are helping the country’s economic development. Furthermore, the industry for electronic transactions has grown rapidly in recent years, expanding financial access (Pea-Assounga and Yao, 2021). Along with their significance in avoiding risks of financial instability, bankruptcy, or failure, particular attention to innovation and investment decisions has increased. It is also possible to generalize the findings of this research to the finance industry. This research thus contributes to the current literature by studying the relationships between internet banking and the decision to invest in banks, as well as by defining the sustainability and competitive advantage mediations that affect investment decisions. We developed a mixed model via a mediation process that links the innovation aspect and the investment decision. Testing our theoretical framework utilizing primary data obtained from 1,500 banks’ employees and customers. We employed the methods of structural equation modeling to evaluate the hypotheses. Data gathered from the respondent was scrutinized and analyzed via the help of SPSS and SmartPLS3.2.8.

The next part examines the theoretical background and hypotheses development. Follows by section three, which deals with methodology and collection of data. Section four lays out the analysis of the results. In the last section, we present the discussion, limits and further investigation directions, and conclusion.

## Literature Review and Hypothesis Development

### Literature Review and Conceptual Framework

Scholars have progressively arrived at the resolution that to get a competitive advantage, organizations have to invest, group, and convey assets (resources), amongst which human capital (employees) and customers are particularly significant (Huo et al., 2015; Ma et al., 2019). Using human capital is frequently more significant than having it for esteem creation (Holcomb et al., 2009), execution (Ndofor et al., 2011), and recuperation from dangers (Ma et al., 2019). Investment implies going through your cash in various monetary resources or foundations for questionable potential compensation consequently and thinking as well about the risk in this procedure.

Nandini (2018) defined investment as a fund commitment made for some positive expectations in return. The expected return from investments can be either capital appreciation, regular income, or a combination of both. Investment decision-taking each day is certainly a fascinating theme; the investment decisions that individuals or institutions such as banks make today are the result of tomorrow’s profits or losses (Nandini, 2018). Nevertheless, not every investment generates benefits as the investment’s decision-makers do not act and behave rationally every time (Velmurugan et al., 2015). Numerous factors affect bank investment decisions such as innovative technologies, stakeholders (customers, employees) satisfaction, knowledge, sustainability, and competitive advantage. Investing can have a substantial impact on banks’ future if they fully understand when, how, and where to invest. Fundamentally, everyone at certain points of life makes investments, by saving or depositing money in a bank, shares, insurance plans, acquisition of equipment, and construction of infrastructures, and every investment implicates risks taken as well (Nandini, 2018).

Antony and Joseph (2017) defined investment decisions as psychological procedures as individuals and organizations settling on choices depending on numerous alternatives that are available. Financial specialists ordinarily embrace investment investigation by utilizing basic examination, specialized examination, and judgment. Technical analysis, fundamental analysis, and instincts depend on the diverse traditional financial hypotheses, which are secured on the standard of rationality (Ogunlusi and Obademi, 2019). The conduct of individuals’ investments is concerned with the option of purchasing small quantities of shares for her or individual account (Jagongo and Mutswenje, 2014). Individuals who invest in securities make this decision. A study by Flor and Hansen (2013) stated that technological advances have an impact on the investment decisions of a company since they significantly influence the cost of investment. Along with demand shocks, they can also influence profitability.

**FIGURE 1:**
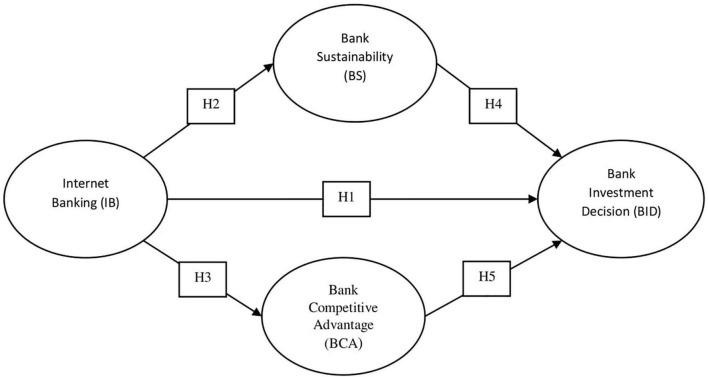
Conceptual framework and the study’s hypotheses.

The empirical works of literature on this subject are broad since the study of corporate investment has been the focal point of financial literacy for the previous numerous periods. Although, these investigations are typically influenced by the impacts of cash flows on investment (Fazzari et al., 1988; Hubbard et al., 1995), the impacts of herding on investment (Grinblatt et al., 1995), corporate investments and CEO confidence, efficiency, and investment management prediction (Goodman et al., 2014), and volatility and investment (Panousi and Papanikolaou, 2012). Our research is different from these works in that we considered the key management concepts namely, sustainability and competitive advantage, and analyze their effects on the banks’ investment decisions (see Figure 1). Also, this research (by goals) integrates the theoretical principles of Theory of Planned Behavior (TPB), Decision Theory (DT), and Resource-Based View Theory (RBVT). The study agrees with Anwar (2018) that the business model (BM) depicts the combination of the specific resources of the company from which the values of customers and organizations can be created. Furthermore, we have emphasized the importance of Decision theory, which demonstrates people’s attitudes and behavior. Decision Theory consists of two variants, namely normative and informative. The normative version posits that an individual must select the action, which maximizes expected utility. The informative version claims that a person always chooses the outcomes that maximize expected utility. In these situations, a decision support system may be very useful in order to reduce the risk of future losses relying on incorrect investment decisions (Peterson, 2017). TPB is an extended version of Reasoned Action Theory (TRA), which has obtained considerable support in empirical investigations on consumer behavior and social psychology (Balushi et al., 2018). TRA’s ability, however, to predict behavior and behavioral intentions when individuals cannot voluntarily control their behavior is limited. We use TPB to study internet banking and investment decision-making in banks. This theory explains and predicts human behavior that is not fully under volitional control where behavioral decisions and proximate behavior are seen as determined primarily by the decision-makers behavioral intentions. RBV theory encourages the hypothesis of the relationship between innovation and company efficiency (Hart and Dowell, 2011).

Particularly, there are four major social consequences of technology-based financial intermediaries. To begin with, Fintech services have substantially improved the efficiency of financial intermediation by providing much cheap, speedier, and more comfortable intermediation via the mobile platforms or the Internet (Buchak et al., 2018; Jagtiani and Lemieux, 2019; Panos and Wilson, 2020; Barbu et al., 2021; Chen et al., 2021). Secondly, for some consumers categories, Fintech may contribute to over-leverage (Boot et al., 2021). Third, in terms of financial inclusions, Fintech services are more likely to serve borrowers with poor credits scores or thin filers (i.e., stockholders without or with few financial transaction records), and their financing activities penetrate zones with very few commercial banks per capita, along with areas in which the local economy is struggling (Jagtiani and Lemieux, 2019; Kou et al., 2021). Fourth, Fintech services have been found to reduce the impact of information asymmetry between the borrowers and the lenders by gathering and using various sorts of soft data for ex-ante credit evaluation for financial customers (Berg et al., 2020; Thakor, 2020). Finally, those Big-Tech-affiliated banks and financial institutions would benefit from the macroeconomic conditions in two different ways: by expanding the overall productivity of companies inside a Big-Tech-driven innovations ecosystem and by exerting competitiveness and contestability on banks and other financial institutions (Breidbach et al., 2019; Duft and Durana, 2020; Barbu et al., 2021; Grant, 2021). Furthermore, a growing number of investigations have demonstrated the implications of the Fintech service industries on diverse micro-aspects of the financial businesses, such as individual investors’ financial behavior during the COVID-19 period (Ionescu, 2021b; Karim et al., 2021), collaborative consumption behavior in the innovation sharing economy (Kuzma et al., 2020), green financial behavior and the transition to a low-carbon economy (Ionescu, 2020a,2021b), and information (Ionescu, 2020a).

Through a variety of routes, digital technology can have both beneficial and unfavorable effects on financial sustainability. For instance, Le et al. (2021) found that the emergence of Fintech credits can operate as a wake-up signal for the banking industry, resulting in a beneficial impact, despite the fact that Fintech credits are more advanced in nations with the less banking systems’ efficiency.

Innovation, either as processes or as outputs, could be defined as a scarce resource that is liable to work as the source of sustained competitive advantage (Kamasak, 2015). The main principle of RBVT is that the foundation of the competitive advantage of an organization resides in the implementation of its wide variety of precious assets and innovation capabilities (comprising technology, procurement, design, distribution, production, and service). This proves that merely those companies that are using their available resources efficiently and have the capacity to innovate, and thus obtain greater performance will gain sustainability and competitive advantage (Zhang et al., 2019). The ability of a firm to restructure valuable and unique resources and consistently promote innovation determines sustainability and competitive advantage (Gürlek and Tuna, 2018). The theoretical line of reasoning of this research, therefore, tends to follow the above three theories that within this potential have also been discussed in previous studies (Warsame and Ireri, 2016; Anwar, 2018; Balushi et al., 2018; Latan et al., 2020). It is notable to admit that the resources of the company will not produce positive results but rather the ability or innovative capabilities of a company to utilize the resources in effective ways. Innovation, for example, includes effective creative practices to consider strategies, consumer expectations, and future predictions of competitor behavior and customer attitudes (Njoroge et al., 2019; Latan et al., 2020). Scholars have suggested that innovation technology seeks the sustainability, competitive advantage, superior performance, and investment decision of the company that can be managed to achieve through the development of new and specific products, the alignment of incentives from various actors, new availability and distribution networks (Acar and Özşahin, 2018; Anwar, 2018; Teece, 2018).

### Hypotheses Development

#### Internet Banking and Bank Investment Decision

The world is now living in a time where, without any thought, the marginal advantages of innovation and technology nevertheless surpass the marginal expenses related to them. Hu and Xie (2016) realized the role of internet banking in terms of increasing the banking sector’s productivity, efficiency, and profitability. Also, some studies have shown that there is a possible link between innovation technologies and investment decisions. For example, Turedi and Zhu (2019) in their study “How to Generate More Value from IT: The Interplay of IT Investment, Decision Making Structure, and Senior Management Involvement in IT Governance,” revealed that the IT decision-making structure mechanisms had a positive moderating effect on the IT investment and organization profitability associations. Božić and Botrić (2017) have argued that the decision to invest properly in innovations is highly complex, on the one hand, inadequate funds, and on the other, based on the variety of innovation paths available to businesses. In a similar vein, they demonstrate that consistent with previous research, a corporation’s decisions to invest in innovations (R&D) tend to increase with its market shares, size, and diversification, as well as with demands-pull and technologies-push influences (Crespi and Zuniga, 2012). Besides that, larger companies invest more in R&D on a per-capita basis but not proportionately, more once the investment decision is made. Moreover, Hashi and Stojčić (2013) highlighted that recipient companies are investing heavily in innovation but also produce lower degrees of innovation outputs, casting doubt on the validity of current national and European Union strategies for innovation subsidization. Another study by Pea-Assounga and Wu (2022) revealed that internet banking is positively and significantly related to bank investment decisions. They further highlighted that constituents of technologies and innovation have diverse effects on investment decisions. We hypothesized the following based on the aforementioned discussions;

*H1: Internet banking significantly and positively influences bank investment decisions.*

#### Internet Banking and Bank Sustainability

Sustainability is concerned with meeting today’s needs without jeopardizing future generations’ ability to meet their own. The ideology of sustainability is built on three pillars: economic, environmental, and social, commonly known as profit, planet, and people.

Njoroge et al. (2019) describe economically sustainable companies as firms with assured cash flows and permanent financial performance. Relying on this meaning, financial sustainability is primarily centered on addressing the needs of shareholders at a given time. Therefore, participating in sustainable measures will rely on the understanding of managers of the anticipated financial benefits (Jassem et al., 2018). Since sustainability investments are associated with risks and unpredictable outcomes (Mullens, 2018), tangible assets, financial capital, and intangible resources such as credibility and innovations must be controlled in order for the company to remain economically sustainable (Njoroge et al., 2019). Economic sustainability is therefore characterized in this investigation as the obligation of banks to undergo economically in various ways, including long-term profitability, sustainable growth, and long-term resource management (Henry et al., 2019). Such obligation is ultimately connected to the policies, activities, and ability of banks to raise and preserve economic performance over time by fulfilling the interests of various stakeholders, such as society, shareholders, and the environment (Evans et al., 2017; Weber, 2017).

Schumpeterian creativity theory suggests that businesses that are continually taking on new variations and creative destruction are more likely to perform better than their competitors (Anwar, 2018). The willingness of banks to innovate is therefore anticipated to be a useful aspect in handling changes in the market climate.

Literature suggests that companies that are unable to innovate run the risks of being side-lined by shifts in legislation, technology, products, competition, and consumer needs (Briglauer et al., 2016). Innovation seeks to improve the effectiveness, profitability, and competitive advantage of companies (Acar and Özşahin, 2018). Thus, corporations need to adapt to changing and demanding market conditions to be competitive (Teece, 2018). Such adaptation can involve, in large part, the proper use of innovative strategies. In other words, the extent to which corporations generate new products, processes, and services which bring value to the markets impacts their economic sustainability (Jassem et al., 2018). In reality, businesses with strong creative strategies appear to search and track their environments continuously while finding new ways to improve their competitive positions (Latan et al., 2020). Studies also show that entrepreneurial, competitive, and disruptive businesses tend to be specialized in environmental scanning, collecting, and processing information (Teece, 2018). Such proven creative practices can be a driving force for businesses to gain economic sustainability (Evans et al., 2017; Njoroge et al., 2019).

*H2: There is a positive significant relationship between internet banking and bank sustainability.*

#### Internet Banking and Bank Competitive Advantage

It is possible to describe innovation as new or creative methods used by the organization or the new goods it produces. The development of new technologies and methods requires innovation. Innovation may be the most significant source of competitive advantage. Innovation attracts rivals. Since innovation offers a business unique benefit (that rivals lack), it can be viewed as the primary source of competitive advantage. Innovation has always been a critical driver of long-term market growth since it leads substantially and positively to value generation, distribution, and sustainability, and assists businesses to identify new business opportunities (Evans et al., 2017). Companies have recently offered sufficient attention to providing new products and replacing existing products by incorporating new features to achieve sustainable competitive advantage (Anwar, 2018). It is ensured that innovation and technology have been shown to be a strong indicators of competitive advantage. Competitors adapt the strategy directly when a company undergoes innovation, so businesses are forced to continually adjust the business model (BM) to achieve a more stable position over their rivals (Anwar, 2018). In contrast to a reactive business, companies with up-to-date and constructive creative services attain higher value and capture great consumer interest in competitive markets. Companies operating in developing and creative markets are looking for competition; innovation is the choice in such a market that makes it easier for companies to achieve a competitive advantage (Anning-Dorson, 2018).

There is a consensus in the literature that all types of innovation can contribute to competitive advantage (Hosseini et al., 2018). Therefore, the following hypothesis will be examined.

*H3: Internet banking has a significant positive effect on bank competitive advantage.*

#### Bank Sustainability and Bank Investment Decision

Banks are generally regarded as having an essential role to achieve the Sustainable Development Goals (SDG) (Louche et al., 2019).

Our investigation is also linked to the literature exploring the impact of sustainability on the decision-making of investors. Dhaliwal et al. (2011) provided a piece of evidence for the US sample that when sustainability reporting (SR) is ensured, the decrease in the cost of equity capital caused by the first-time sustainability is estimated two times greater. Casey and Grenier (2015) broaden this review and have clear overall results by applying a US study. Besides that, they notice that if an auditor offers sustainability, the decrease in the cost of equity capital is much more prominent.

Cheng et al. (2015) show that SA improves the willingness of equity providers to invest primarily when the sustainability knowledge mentioned plays a more significant role in the strategies of the company. A study by Jassem et al. (2018) revealed that Sustainability Balanced Scorecard (SBSC) positively influences the decision-making of environmental investment. Similarly, Steinmeier and Stich (2019) argued that Sustainability Assurance (SA) increases the internal knowledge set available to decision-making managers, contributing in turn to an improvement in the efficiency of Sustainability Investment (SI). They added that in the increasingly important area of corporate sustainability, SA assists or forces managers to recognize favorable SI prospects and enhance managerial investment decisions.

Financial services can encourage or impede (non-)sustainable activities of governments, businesses, and individuals because of their intermediary role, and can also activate systemic changes in society (Louche et al., 2019). Therefore, these offerings are called transformative services that affect society and the environment in a very specific way. Companies may typically adopt social practices (i.e., health management, corporate volunteering), sustainable practices (i.e., energy savings), and external practices, as briefly mentioned above (i.e., donations). The same is true for banks, but activities that are embedded in the core businesses of these organizations have far more comprehensive consequences. Another investigation by Zimmermann (2019) stated that within the peripheral areas of their activities, banks could facilitate external sustainability initiatives in terms of resources or materials. The author added that banks always consider aspects of sustainability as they undertake investment decisions. Based on the above perspectives, we assume that:

*H4: There is a significant relationship between bank sustainability and bank investment decision.*

#### Relationship Between Bank Competitive Advantage and Bank Investment Decision

Competitive pressures are present in almost every industry. These pressures lower returns on capital and could lower future investment returns. Finding companies with sustainable competitive advantages can aid investors in generating higher investment returns (Latan et al., 2020).

An investigation by Briglauer et al. (2016) demonstrated that service-based competitiveness does not seem to have a substantial effect on incumbents’ and entrants’ investment decisions. Nevertheless, with regard to the later phase of markets’ liberalization, competition dependent on services has a negative effect on the investment of entrants. Another work by Laksmana and Yang (2015) finds that competitiveness risky investments invest in a risky investments. They also indicate the management of competition disciplines on the use of free cash flows (FCFs). Generally, their findings provide evidence for the disciplinary role of competition in the market of products and organizations’ investment decisions.

According to the above discussion, we hypothesized;

*H5: Bank Competitive Advantage has a positive significant relationship with Bank investment decisions.*

#### The Mediating Effects of Bank Sustainability and Bank Competitive Advantage on the Relationship Between Internet Banking and Bank Investment Decision

The profits of a firm, contrary to common belief, are not the outcome of the services and products it offers. In addition, when deciding whether to invest, successful investors do not only rely on an organization’s earnings. The profits of a company are the product of its sustainability and competitive benefits. Consequently, successful investment managers concentrate on whether the sustainability and competitive advantages of a firm would allow it to protect and increase profits (Anwar, 2018). The investors desire to possess great business returns that could generate those sustainable benefree-market is an issue since free market competition attempts to urge the returns down (Xin and Choudhary, 2019). Proven empirical evidence that certain organizations have seen sustainable returns on capital invested over long periods, emerging a list of attributes, which lead to sustainable returns on capital, would enhance future profits and investment choices (Latan et al., 2020). Decision Theory consists of two variants, namely normative and informative. The normative version posits that an individual must select the action, which maximizes expected utility. The informative version claims that a person always chooses the outcomes that maximize expected utility. In these situations, a decision support system may be very useful in order to reduce the risk of future losses relying on incorrect investment decisions (Peterson, 2017).

Resource-Based View Theory (RBVT) explains that capital (resources) ownership is valuable, difficult to duplicate, rare, and irreplaceable (Grant, 1991). This suggests that businesses’ assets are entrusted with a critical role in assisting organizations in achieving better performance, acquiring a competitive edge, and ensuring long-term sustainability (Nevo and Wade, 2010). Finally, TPB as an extended version of the Theory of Reasoned Action (TRA) posits that individuals undertake reasoned and logical decisions to involve in specific behaviors by assessing the available information they have (Balushi et al., 2018). The performance of a behavior is determined by the individual’s intention to engage in it (influenced by the value the individual places on the behavior, the ease with which it can be performed, and the views of significant others) and the perception that the behavior is within his/her control. In Reasoned Action (RA) a TPB model based on social support, attitudes, and self-efficacy. A behavioral intention represents an individual’s commitment to acting and is itself the outcome of a combination of several variables.

From the foregoing theoretical underpinnings and practical observations, as well as the fact that online banking services are a precondition for innovative technologies and bank investment decisions, bank sustainability and bank competitive advantage can help to evaluate bank investment decision-making, therefore we assume that BS and BCA would mediate the link between internet banking and BID. The resulting hypotheses will be tested based on this assumption:

*H6: Bank sustainability would mediate the relationship between internet banking and Bank investment decision.*

*H7: bank competitive advantage can mediate the link between IB and Bank investment decisions.*

## Methodology and Data

This research employs a methodological approach that emphasizes the use of quantitative data collection and analysis tools. The quantitative factors were measured using a questionnaire survey. The objective of this research is to assess the effects of online banking as well as bank sustainability and competitive advantage mediation on bank investment decisions.

A suitable research design is the primary methodological concern when examining the theoretical model. The scholars considered employees’ and customers’ points of view in this research for several reasons. Firstly, employees are very important to organizational innovation as they are responsible for interacting, modifying, and developing ideas. Similarly, to be innovative, organizations must manage and improve the internal environment that supports the characteristics of employees’ innovative behavior (Pea-Assounga and Yao, 2021). Secondly, customers are also extremely important in any organization, as to survive in today’s innovative and competitive world, a firm should improve customers’ services and consider their requests and complaints seriously. In addition, to criticize earlier research, Homburg et al. (2009) present convincing evidence of the necessity of taking into account both customers and employees in a single study. It is vital to use a significant sample size to test the theoretical model of our research, in which the level of interactions between staff and customers is an important component of the customer’s entire experiences with the services provider in the business (Jeon and Choi, 2012). Conclusively, the banking industry, particularly the interactions between consumers and workers, is a great example, as these interactions (between branch personnel and clients) do not need either individual to become engaged in task-related. On the other hand, the outcomes of the customer interaction and employee’s interaction with the customer are critical to the efficient delivery of the banks’ services and products, as well as a great customer experience (Brown and Lam, 2008). Employees in the banking system are in charge of managing and communicating with consumers on a daily basis. Moreover, the companies that actively aim to create an internal climate that sees employees’ demands as equally important as customers’ requirements (Gounaris and Boukis, 2013) should justifiably expect greater levels of customer and employee loyalty in return (Gounaris and Boukis, 2013; Mainardes et al., 2019). As a result of this feature of our study’s environment, we expected to consider banks’ internal and external aspects to provide insights analysis. Therefore, we decide to match staff and individual consumers across the 11 commercial banks’ data collection, which was a prerequisite for evaluating our research hypotheses. In a similar line, some researchers have adapted and considered both staff and customers in a single study (Jeon and Choi, 2012; Jung and Yoon, 2013; Jha et al., 2017; YuSheng and Ibrahim, 2020). Conclusively, this enables us to conduct a single study that includes both staff and customers.

To do this, we first secured senior management consent from 11 active commercial banks, after which we approached individual branch managers and asked for their help with the study. In total, 55 branches agreed to take part in the research. We targeted the collection of data on 1,200 clients and 600 workers from 11 licensed commercial banks in the country based on the information available from the banks. On one hand, the surveys were shared with 600 employees from 11 institutions in our sample, which included 55 branches. The 500 employee questionnaires with valid responses have been obtained during the data collection, resulting in an approximately 83 percent employee response rate. The study enlisted the participation of an average of nine (9) employees in each branch. On the other hand, we utilized proportionate stratified random sampling once more to verify that the number of consumers in the sample across the two major cities was proportional to the percentage of total customers in the 11 banks. We collected 1,000 survey questionnaire responses from clients out of 1,200 initial targets using this approach, resulting in an 83 percent overall customer response rate. The available banking industry archival data provided to us correspond to customer engagements (i.e., customers’ participation and engagement in banking services and products) before and during the study period.

To sum up, we collected 1,000 and 500 usable questionnaires from banks’ customers and workers, respectively. Numerous rules of thumb recommend that a “50/20” or a “100/10” percentage is satisfactory (Hox et al., 2010). In our situation, the proportion is in the middle of these two ranges, which is consistent with the findings of numerous social science studies (Giardini and Frese, 2008; Jung and Yoon, 2013; YuSheng and Ibrahim, 2020).

The data was collected from the distinct work units of 11 banking institutions that are now operating in Brazzaville and Pointe-Noire, Republic of Congo, the two cities that have the majority of the banks’ branches. The completed survey is comprised of close-ended questions. To ensure correct interpretation and transmission of the items’ intended implications, the survey instruments were first translated into French and then retranslated into English. The data were collected from November 2019 to June 2020. Additionally, the study used a “Likert scale” ranging from 1 to 5, with 1 indicating “strongly disagree” and 5 indicating “strongly agree,” as well as pertinent questionnaires from other scholars who conducted similar studies.

## Constructs Measurements

The measurements employed in this research were derived from earlier studies and tailored for this study. The items of internet banking have been undertaken by Rahi et al. (2019); and (Wu et al., 2012), the items for bank sustainability were adopted from Njoroge et al. (2019), while the items for bank competitive advantage were taken from Anwar (2018) and finally items for bank investment decision are adopted from Ogunlusi and Obademi (2019). The questionnaires for research items can be found in Table A1. Datasets collected from the participants have been scrutinized and analyzed with the help of SmartPLS and SPSS. Demographic analysis has been done through SPSS, while the hypotheses testing and other tests were carried out in both SPSS and SmartPLS.

### Justification of the Partial Least Square-Structural Equation Modeling Model Selection

Structural equation modeling (SEM) is a method that concurrently solves worldwide various social sciences problems and examines factors’ relationships *via* diagrams. SEM has also been used to analyze the model’s fit using approaches like path analysis, factor analysis, and regression analysis (Kline, 2016). This approach appears to be more statistically accurate than other approaches (Hair et al., 2017). SEM as a testing cause and effect model using latent variables has become a standard analytical procedure. In business research, partial least square path models (PLSPM) are frequently utilized as a composite-based estimator to study structural equation models with latent variables. Herman Wold first created this technique in the 1970s as an alternative to covariance-based structural equation modeling (CB SEM) (Joreskog, 1982; Wold, 1982).

Partial least square-structural equation modeling and CB SEM are logically different (Hair et al., 2014; Dash and Paul, 2021). For theoretical testing and validation, the CB SEM technique is ideal. PLS-SEM is the most effective method for making predictions and constructing theories. However, increasing the amount of explained variance independent constructs is also a goal. CB-SEM is the best choice for testing and confirming existing hypotheses. PLS-SEM, on the other hand, is excellent for theory development and forecasting (Dash and Paul, 2021). For acquiring complete knowledge about the determinants of consumer satisfaction, brand image, or corporate reputations, PLS-SEM is a great approach. PLS-SEM is the same as CB-SEM for many scientists (Hair et al., 2021). In business, empirical approaches are utilized for explanation and prediction (Sarstedt et al., 2014). The application of CB-SEM frequently overlooks a crucial goal of empirical research: prediction (Hair et al., 2021). PLS-SEM, which tries to anticipate endogenous latent variables, is the solution to this basic issue. In comparison to CB-SEM, PLS-SEM has a few advantages. Many empirical analysts just acknowledge the variables’ distributional assumptions. Indeed, the majority of empirical data in social sciences and business is non-normal (Hair et al., 2014, 2021; Sarstedt et al., 2014; Dash and Paul, 2021). The majority of CB-SEM applications overlook the common violations of this technique’s criteria. As PLS-SEM does not require these strict “distributional assumptions,” it is a viable alternative to CB-SEM. Therefore, consistent PLS-SEM is relevant and suited for our study since it focuses on assessing a theoretical framework from the viewpoint of confirmation, and reflective constructs, as well as multiple models/relationships (Dijkstra and Henseler, 2015).

### Measurements Scales Reliability and Validity

The composite reliability and Cronbach’s alpha coefficients were calculated to assess the constructs’ reliability.

Cronbach’s alpha is a metric for determining the consistency or reliability of a set of scale or test items. As per the findings, Cronbach’s Alpha (CA) ranges from 0.863 to 0.875 which is above the 0.7 acceptancy value. In addition, all the lower bounds of the Cronbach’s Alpha confidence interval are greater than 0.7, indicating that internal consistency is verified (Trinchera et al., 2018). The interval confidence values were determined in SPSS under the function intraclass correlation coefficients (Baumgartner and Chung, 2001). To ensure the validity of Cronbach’s Alpha estimates and confidence interval values, we have compared the values with the ones obtained from consistent PLS; the Cronbach’s Alpha outcomes were the same. Furthermore, composite reliability (CR) ranges from 0.863 to 0.885, and this is higher than 0.7 and indicates that the variables are extremely reliable (Hair et al., 2019).

The values of the average variance extracted (AVE) have been examined to ensure convergence validity. The results demonstrate that the AVE’s values for this study range from 0.509 to 0.663, which is acceptable and higher than the minimum required degree of 0.5. Table 1 displays all validity and reliability results. Additionally, the criterion of Fornell-Larcker was surveyed for assessing the discriminant validity of estimation models. “Fornell-Larcker criterion” necessitates that the AVE’s squares roots must be greater than any other correlations of the constructs’ relationships with other users in the hypothetical model (Hair et al., 2017). The results of this examination satisfy this standard and the complete estimation of the Fornell-Larcker criterion is reported in Table 1.

**TABLE 1:** Constructs reliability and validity, and discriminant validity.

Cronbach’s alpha					Composite reliability (CR)	Average variance extracted (AVE)	Discriminant validity (Fornell-Larcher criterion)			
				
	Coefficient	95% CI		*P*-value			BCA	BID	BS	IB
BCA	0.875	0.865	0.885	0.000	0.885	0.663	**0.814**			
BID	0.859	0.848	0.870	0.000	0.860	0.509	0.684	**0.713**		
BS	0.847	0.834	0.859	0.000	0.861	0.615	0.560	0.608	**0.784**	
IB	0.863	0.852	0.874	0.000	0.863	0.514	0.479	0.466	0.436	**0.717**

*Underlined and bold Values are the squares roots of AVE.*

We assessed descriptive statistics of the items that comprised means, standard deviations, kurtosis, skewness, and the VIF. The results in Table 2 show that mean values are between 3.198 and 3.45. Most importantly, the VIF values are all less than three, implying that there is no collinearity issue (Hair et al., 2019).

**TABLE 2:** Descriptive statistics of indicators.

Items	Mean	Median	Min	Max	Standard deviation	Kurtosis	Skewness	VIF
BCA1	3.202	3	1	5	1.033	−0.506	−0.259	2.572
BCA2	3.198	3	1	5	1.097	−0.633	−0.243	2.672
BCA3	3.201	3	1	5	1.124	−0.690	−0.237	2.345
BCA4	3.203	3	1	5	1.135	−0.717	−0.232	2.030
BID1	3.458	4	1	5	1.062	−0.473	−0.381	2.295
BID2	3.451	4	1	5	1.066	−0.556	−0.326	2.570
BID3	3.227	3	1	5	1.153	−0.744	−0.263	2.173
BID4	3.239	3	1	5	1.147	−0.740	−0.254	2.209
BID5	3.231	3	1	5	1.110	−0.658	−0.291	2.245
BID6	3.493	4	1	5	1.054	−0.126	−0.582	1.808
BS1	3.431	3	1	5	1.002	−0.239	−0.346	2.365
BS2	3.437	4	1	5	1.097	−0.410	−0.409	1.971
BS3	3.391	3	1	5	1.114	−0.494	−0.365	2.054
BS4	3.415	3	1	5	1.105	−0.507	−0.356	2.003
IB1	3.315	3	1	5	1.177	−0.677	−0.391	2.730
IB2	3.255	3	1	5	1.135	−0.739	−0.244	1.288
IB3	3.214	3	1	5	1.137	−0.725	−0.246	2.661
IB4	3.309	3	1	5	1.179	−0.708	−0.386	2.485
IB5	3.247	3	1	5	1.149	−0.799	−0.224	2.220
IB6	3.292	3	1	5	1.182	−0.739	−0.359	1.278

The other results revealed both kurtosis and skewness values are relatively small and negative. Particularly, the negative values of the skewness imply that the data are skewed left while the kurtosis negative values indicate a “light-tailed” distribution. We also evaluated the correlation of the indicator in our research. The findings indicated a substantial and positive correlation between the study’s items, with values, ranging from 0.164 to 0.789. Table 3 demonstrates that no correlation value is above the 0.9 thresholds, implying that there is no multicollinearity issue between the research items (Lowry and Gaskin, 2014).

**TABLE 3:** Indicators correlation matrix.

	BCA1	BCA2	BCA3	BCA4	BID1	BID2	BID3	BID4	BID5	BID6	BS1	BS2	BS3	BS4	IB1	IB2	IB3	IB4	IB5	IB6
BCA1	1.000																			
BCA2	0.789**	1.000																		
BCA3	0.755**	0.572**	1.000																	
BCA4	0.709**	0.529**	0.504**	1.000																
BID1	0.535**	0.416**	0.414**	0.373**	1.000															
BID2	0.450**	0.336**	0.335**	0.293**	0.777**	1.000														
BID3	0.452**	0.348**	0.324**	0.292**	0.391**	0.360**	1.000													
BID4	0.468**	0.363**	0.339**	0.410**	0.399**	0.301**	0.580**	1.000												
BID5	0.547**	0.435**	0.409**	0.374**	0.475**	0.399**	0.726**	0.731**	1.000											
BID6	0.466**	0.367**	0.340**	0.313**	0.622**	0.468**	0.422**	0.427**	0.499**	1.000										
BS1	0.574**	0.469**	0.441**	0.365**	0.474**	0.362**	0.417**	0.439**	0.508**	0.441**	1.000									
BS2	0.413**	0.324**	0.301**	0.233**	0.306**	0.278**	0.396**	0.285**	0.344**	0.279**	0.700**	1.000								
BS3	0.387**	0.300**	0.279**	0.212**	0.330**	0.270**	0.272**	0.273**	0.353**	0.294**	0.712**	0.461**	1.000							
BS4	0.403**	0.315**	0.293**	0.225**	0.306**	0.205**	0.252**	0.290**	0.329**	0.292**	0.705**	0.484**	0.464**	1.000						
IB1	0.329**	0.265**	0.232**	0.217**	0.267**	0.222**	0.149**	0.162**	0.227**	0.283**	0.291**	0.170**	0.226**	0.211**	1.000					
IB2	0.380**	0.302**	0.287**	0.243**	0.272**	0.219**	0.228**	0.233**	0.277**	0.323**	0.353**	0.225**	0.242**	0.264**	0.367**	1.000				
IB3	0.336**	0.261**	0.246**	0.203**	0.233**	0.179**	0.226**	0.217**	0.261**	0.291**	0.302**	0.223**	0.195**	0.266**	0.339**	0.779**	1.000			
IB4	0.372**	0.302**	0.270**	0.255**	0.326**	0.246**	0.183**	0.196**	0.226**	0.349**	0.333**	0.209**	0.220**	0.220**	0.685**	0.415**	0.359**	1.000		
IB5	0.316**	0.243**	0.229**	0.186**	0.225**	0.195**	0.175**	0.179**	0.251**	0.264**	0.285**	0.164**	0.240**	0.185**	0.371**	0.719**	0.645**	0.373**	1.000	
IB6	0.351**	0.286**	0.252**	0.237**	0.272**	0.250**	0.173**	0.186**	0.250**	0.298**	0.311**	0.190**	0.265**	0.200**	0.778**	0.393**	0.336**	0.746**	0.394**	1.000

N = 1,500, ** “Correlation is significant at the 0.01 level (2-tailed).”

### Common Methods of Bias Test

As both dependent and independent factors were quantified using the same questionnaires, it is critical to conduct a common technique of bias test (Lowry and Gaskin, 2014). The study’s constructs Harman’s single factor test was used. The results suggested that the single factor describes around 39% of the total variance in the model and is less than the crucial level of 50%, indicating that there is no common method bias in the variables (Lowry and Gaskin, 2014).

**FIGURE 2:**
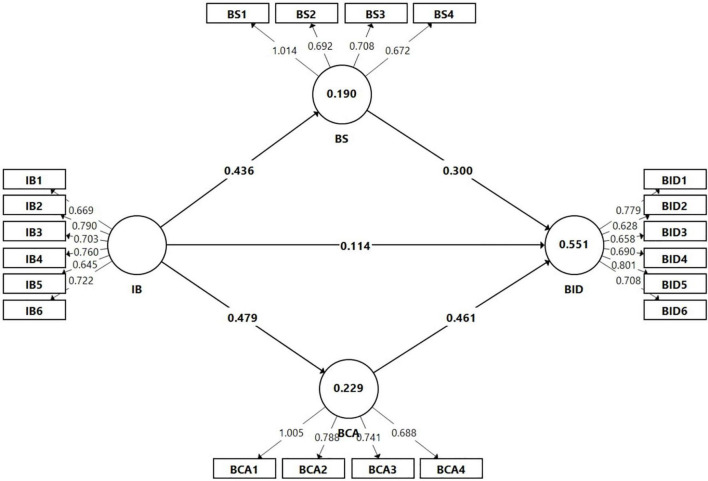
Structural model.

The fit indices of the measurement model, such as the SRMR and NFI have been also assessed, and the values are 0.064 and 0.971. This indicates that the fit indices were within acceptable limits. In addition, the item loadings were further examined, and the values are varying from 0.628 to 1.014 (see Figure 2), which are acceptable (Hair et al., 2019). The indicators’ loadings values that are equal to or greater than 0.5 and statistically significant are considered relevant for the analysis (Ringle et al., 2015; Hair et al., 2019). As a result, the measurements of scales were deemed appropriate for use in subsequent analysis. The fit indices and item loadings were obtained with consistent PLS.

## Results

The questionnaires comprise the respondents’ profiles: gender, age, educational level, and Income per month. The results from Table 4 below depict that 752 respondents were males representing 0.1%, and 748 respondents representing 49.9% were females, and 400 of the participants representing 26.7% were between the ages of “18–25 years”. The 503 respondents representing 33.5% were between the ages of “26–35 years”; 356 respondents representing 23.7% were between the ages of “36–45 years”; 241 respondents representing 16.7% were above the “age of 46 years”. The outcomes in Table 4 also demonstrate that 123 of the respondents representing 8.2% are from the “Basic level”; 240 respondents representing 16.0% is from the “High school” whiles 361 respondent representing 24.1% are from the “diploma level”; 511 of the respondents representing 34.1% are from the “undergraduate degree” whiles 265 of the respondents representing 17.7% are from the “graduate level.” In addition, Table 4 depicts the related statistics to income per month (in thousands of FCFA, XAF) of the respondents. The results show that 141 of the respondents representing 9.4% receive up to 90 thousand income per month. Approximately 24% of respondents gain an income between 91 to 150 thousands monthly whiles 314 respondents representing 21% of their income belong to the group 151–200 thousand; 216 of the respondents representing approximately 15% receive a monthly income ranging from 201 to 300 thousand whiles 267 of the respondents representing 18% their income per month is between 301 and 499 thousand. Finally, the remaining approximately 14% of the respondents have a monthly income of 500 thousand and above.

**TABLE 4:** Demographics of participants.

	Participants (No. = 1,500)					

	Frequency			Percent		
		
	Total	Employees	Customers	Total	Employees	Customers
**Gender**						
Male	752	253	499	50.1	50.6	49.9
Female	748	247	501	49.9	49.4	50.1
**Age**						
18–25 years	400	143	257	26.7	28.6	25.7
26–35 years	503	165	338	33.5	33	33.8
36–45 years	356	120	236	23.7	24	23.6
46 and above	241	72	169	16.1	14.4	16.9
**Educational level**						
Basic level	123	36	87	8.1	7.2	8.7
High school	240	72	168	16.0	14.4	16.8
Diploma	361	122	239	24.1	24.4	23.9
Undergraduate	511	187	324	34.1	37.4	32.4
Graduate (Master’s or Ph.D.)	265	83	182	17.7	16.6	18.2
**Income per month (in XAF)**						
Up to 90,000	141	44	97	9.4	8.8	9.7
91,000–150,000	356	118	238	23.7	23.6	23.8
151,000–200,000	314	99	215	20.9	19.8	21.5
201,000–300,000	216	80	136	14.4	16	13.6
301,000–499,000	267	90	177	17.8	18	17.7
500,000 and above	206	69	137	13.8	13.8	13.7
No. of participants = 1,500						

We conducted the assumption tests to check that the equal variances and equal means of the two groups (employees and customers), e.g., are not significantly distinct. The Test of Homogeneity of Variance, often known as Levene’s test was used to determine whether the population included in the sample frame is homogenous (Derrick et al., 2018). According to the findings, there are no statistically significant differences between the two groups; Levene’s Statistic for endogenous construct, bank investment decision (BID), [*F*_(1, 1498)_ = 0004; *P* = 0.951], this implies that the test of equality of variances was not violated. The Brown-Forsythe Test, on the other hand, with values of Statistic = 0.003; *P* = 0.953 for the BID validated the robustness of Levene’s test (Gastwirth et al., 2009). Also, the means values of the two groups were 20.09 and 20.1 for employees and customers, respectively. This means that the assumptions of homogeneity of variances and equal means are verified (e.g., not violated) in our research.

### Analysis of Hypotheses

We employed consistent partial least square (PLSc) to conduct the SEM, as recommended by Dijkstra and Henseler (2015) that the PLSc estimates indicators’ loadings and path coefficients consistently, and the outcomes are similar to the ones from CB SEM. Using the statistical tools SmartPLS and SPSS, we tested the hypotheses on the effects of IB on BID. The results indicated a substantial correlation between the endogenous constructs and the predictors’ factors.

### Direct Effects of Internet Banking on Bank Investment Decision, Bank Sustainability, and Bank Competitive Advantage, as Well as the Effects of Bank Sustainability and Bank Competitive Advantage on Bank Investment Decision

To evaluate direct effect of internet banking (IB), bank sustainability (BS), and bank competitive advantage (BCA) on bank investment decision, we employed a consistent PLS structural equation modeling. Internet banking was found to have standardized coefficients (see Table 5) that were significantly and positively associated with bank investment decision, bank sustainability, and bank competitive advantage. The results revealed that these coefficients were IB - > BID (β = 0.114; *t* = 3.69; *p* < 0.01), IB - > BS (β = 0.436; *t* = 15.112; *p* < 0.01) and IB - > BCA (β = 0.479; *t* =0 18.542; *p* < 0.01), respectively. Therefore, the hypotheses (H1, H2, and H3) were supported.

**TABLE 5:** Hypotheses testing, standardized coefficients (PLSc-SEM).

Hypothesis	Path	Coefficient	St. Error	T-statistic	*P*-value	95% CI		*f* ^2^	VIF	Action
H1	IB - > BID	0.114	0.031	3.690	0.000	0.030	0.200	0.021	1.371	Confirmed
H2	IB - > BS	0.436	0.029	15.112	0.000	0.350	0.511	0.235	1.000	Confirmed
H3	IB - > BCA	0.479	0.026	18.542	0.000	0.407	0.545	0.297	1.000	Confirmed
H4	BS - > BID	0.300	0.031	9.734	0.000	0.215	0.390	0.130	1.539	Confirmed
H5	BCA - > BID	0.461	0.032	14.571	0.000	0.374	0.540	0.292	1.617	Confirmed

*BCA, Bank Competitive Advantage; BID, Bank Investment Decision; BS, Bank Sustainability; IB, Internet Banking.*

The outcomes in Table 5 also demonstrated that both bank sustainability and bank competitive advantage have statistically positive and significant effects on BID with the coefficients BS - > BID (β = 0.3; *t* = 9.734; *p* < 0.01) and BCA - > BID (β = 0.461; *t* = 14.571; *p* < 0.01). Conclusively, H4 and H5 are also accepted. The mediation analysis is used to test the other hypotheses.

### Mediation Analysis

The term “mediation” refers to the study of how a third variable influences the relationship between other two different variables. A mediation analysis was performed as part of this study.

Table 6 shows the indirect effects of IB on banks’ investment decisions, these effects are mediated via bank sustainability and bank competitive advantage factors.

**TABLE 6:** Tests of mediation (“Direct, indirect, and total effects, PLSc outputs”).

Hypothesis	Direct effects	Std. Coef.	St. Err.	Z	P > z	(95% C)	Decision
	IB - > BID	0.114	0.031	3.690	0.000	0.030–0.200	
	**Indirect effects**						
H6	IB - > BS - > BID	0.131	0.016	8.110	0.000	0.092–0.181	Partly supported
H7	IB - > BCA - > BID	0.221	0.019	11.721	0.000	0.170–0.276	Partly supported
	**Total effects**						
	IB - > BID	0.466	0.030	15.633	0.000	0.383–0.539	

In details, Table 6 indicates that the “indirect relationship between” internet banking and bank investments decisions via bank sustainability is statistically positive and significant (IB - > BS - > BID; β = 0.131; *t* = 8.11; *p* < 0.01). Likewise, the mediating effect of bank competitive advantage on the link between internet banking and bank investment decision is as IB - > BCA - > BID (β = 0.221; *t* = 11.721; *p* < 0.01). Along the same lines, a significant statistical mediation effect may be determined, implying that hypotheses (“H6 and H7”) are also partially supported. Comparably, BCA depicts greater significant direct and indirect effects on BID than BS (see Tables 5, 6 and Figure 2).

The outcomes in Tables 6, 7 show that both “direct and indirect effects of internet banking” on BID are positively and statistically significant, designating that bank sustainability (BS) and bank competitive advantage (BCA) jointly and partially mediated the effect of internet banking (Nitzl et al., 2016).

**TABLE 7:** Mediation analysis using Sobel test (SPSS syntax) and variance accounted for (VAF).

Path	Effect	SE	95% CI	
(y1) IB - > BS (Direct effect)	0.250	0.018	0.215	0.284
(y2) BS - > BID (Direct effect)	0.610	0.036	0.541	0.680
(y3) IB - > BID (Direct effect)	0.222	0.025	0.174	0.271
(y1*y2) IB - > BS - > BID (Indirect effect)	0.153	0.013	0.128	0.180
(y3 +y1*y2) IB - > BID (Total effect)	0.375			
Variance Accounted For (VAF)	0.408			
(y4) IB - > BCA (Direct effect)	0.290	0.016	0.258	0.322
(y5) BCA - > BID (Direct effect)	0.701	0.030	0.642	0.760
(y6) IB - > BID (Direct effect)	0.172	0.021	0.130	0.213
(y4*y5) IB - > BCA - > BID (Indirect effect)	0.203	0.015	0.174	0.233
(y6 +y4*y5) IB - > BID (Total effect)	0.375			
Variance Accounted For (VAF)	0.541			

The Variance Accounted For (VAF) value can be used to assess the strength of the mediation analysis. Also, as suggested by Shrout and Bolger (2002) and Ditlevsen et al. (2005), when the direct path effect sign is the same and strong as the indirect path effect, the scholar should evaluate the strength of the mediation analysis by employing the ratio effect (i.e., VAF). To this end, the VAF value was assessed.

Bank sustainability and BCA are the intermediates in this study, as per the literature and postulated model. In the instance of PLS-SEM, the variance accounted for (VAF) approach was also utilized to investigate the mediating effects, since it is one of the greatest ways of examining the mediation effects (Hair et al., 2014). The VAF value is the ratio of the indirect effects’ coefficient to the total effects.

Full mediation is defined as a VAF value greater than 0.8; partial mediation is defined as a VAF value ranging from 0.2 to 0.8; no mediation is defined as a VAF value is below 0.2 (Hair et al., 2014). Table 7 shows the mediation paths as well as the VAF values. These results were obtained from SPSS using “Sobel test SPSS syntax” (Preacher and Hayes, 2004). The researchers have appropriately assessed the relationships and presented their findings, according to the mediation analysis and its outcomes. With a VAF value of 0.408, the effects of IB on BID with the mediating effects of BS are shown to be substantial, confirming the partial mediation effects between IB and BID. As a result, H6 is partially supported. The second mediation path shows that BCA is mediating the interaction between IB and BID. With a VAF value of 0.541, the outcomes of mediation analysis are significant and positive, indicating that there is a partial mediation influence on the relationship. As a result, the study partially confirms the H7. In comparison with the results from consistent PLS-SEM, it is necessary to note that there is no significant difference between the two outcomes as both methods confirmed the presence of partial mediation.

## Discussion

In this study, we notice that constituents of innovation, sustainability, and competitive advantage affect the bank investment decisions. Our findings confirm the argument appointed by previous studies that investment decision depends on various factors (Božić and Botrić, 2017; Turedi and Zhu, 2019). In addition, we found that diverse constituents of technologies and innovation have different effects on bank investment decisions. Particularly, IB, directly and indirectly, exerts a positive impact on banks’ investment decisions. This result is in line with the argument of Hashi and Stojčić (2013) which states that firms’ decisions depend on the knowledge accrued from innovations and the usage of resources. The finding is also consistent with Pea-Assounga and Wu (2022) who found a positive and significant link between internet banking and BID. These findings show that simply focusing on sustainability and competitiveness through innovative services and activities is insufficient to ensure the benefits anticipated from bank decisions. Furthermore, stakeholders may play the most valuable role in promoting bank investment decisions. This could be due to the “relationship-oriented” cultural context in the commercial environment of banks that exemplifies interpersonal and social contractual relationships. Businesses with important technologies and innovation are able to create effective relationships with different stakeholders in such an area, which can generate more market opportunities and therefore lead to improved outputs. Moreover, this finding is in line with Flor and Hansen (2013) who found that technological advances affect a company’s investment decisions. Shortly, these findings add to the current pieces of literature on technologies and innovation by exposing various fundamental mechanisms by which constituents of innovation contribute to improving the way banks make their choices. In view of its strategic goals for various performance results, they also contribute to the management literature by recommending which innovation aspect organizations can devote greater sustainable and competitive resources. In addition, our research findings are in line with Carboni and Medda (2019) who found that innovation activities are positively and significantly related to investment decisions. This implies that the relationship between innovation and investment decisions is critical for a company’s growth, especially given the close relationship between the accumulation of tangible capital and technological advancement. Furthermore, our findings are also consistent with Dwivedi et al. (2021) who revealed that Fintech adoption positively and significantly influences the competitiveness and performance of banks in the UAE.

Secondly, our results affirm the mediating roles of sustainability and competitive advantage in the IB-BID relationship, like this, reinforcing the view that multiple factors can mediate the effect of internet banking on bank investments decision (Shahwan, 2018). This finding is also consistent with Zhang et al. (2019) who revealed that technological innovation and management innovation have positive and significant effects on sustainability and organizational performance. In the same vein, our findings are in line with Gürlek and Tuna (2018) who highlighted that green innovation is positively related to competitive advantage. Nevertheless, our study is one of the first to investigate the roles of sustainability and competitive advantage as mediators. It provides an alternate reason for the IB-investment decision relationship. In particular, the impact of IB on BID is partially mediated by both sustainability and competitive advantage. As shown in Tables 5–7 and Figure 2, competitive advantage has a greater impact than sustainability in influencing bank investments decision. These findings contribute to currently available innovation and management pieces of literature and can serve as guidelines for businesses in developing a comprehensive innovation process to enhance IB, stakeholder loyalty, and bank investment decisions. Thirdly, while some prior studies have concentrated on innovative activities and firm investment decisions, innovation investment decisions or investment actions as the main constructs (Božić and Botrić, 2017; Nandini, 2018; Shahwan, 2018; Carboni and Medda, 2019). This research focuses on sustainability and competitive advantage, the two primary dimensions of environmental management, and shows that they are positively influenced by IB. Our research indicates positive relationships between IB, sustainability, competitive advantage, and investment decisions. These findings shed new light on structural processes that facilitate bank investment decisions.

### Limitations and Potential Avenues for Future Research

This study has some limits, which can be discussed by further studies. First, data have been gathered in the Republic of Congo, thus providing difficulties to generalize the context. Further works should test whether the constructs employed in the present research may also be examined in other countries. Second, information about how techniques emerge is not covered by the data. Future research may explore the changes in trends. Third, this investigation is not illustrative with concern to the distribution of banks in Congo because its foundation in data is limited only to two main cities namely Pointe-Noire and Brazzaville. Therefore, it is an attractive issue for scholars to shed light on in all cities in Congo or other countries in the World. Such information would allow an investigation of the examined determinants of firm innovation, sustainability, competitive advantage, and investment decisions and an analysis of proposed relationships. Particularly, banks’ patterns for innovation services, competition, sustainability, investment decisions, and the ways in which the decision process is taken, may play a vital role in sustainable benefits. Additional research directions comprise the absence of moderator constructs such as internet security, gender, perceived risk, and investment risk in this investigation. In order to strengthen this research results, further study may analyze our conceptual framework with a moderation effect. Scholars may also examine the reasons financial institutions do not consider the significance of environmental and social aspects in maintainable growth and thus, forgo sustainable practices implementations. In addition, the sustainability strategies typology assists in better measurements of the specific effects of sustainability practices on behaviors and clients’ attitudes. The examination of this association between clients’ responses and corporate sustainability also unlocks possibilities for future investigations. Another road for future research is that we have not hypothesized the relationship between sustainability and competitive advantage, and the links between sustainability, competitive advantage, and financial performance. Therefore, future studies may adopt our research framework and include the above-mentioned relationships.

## Conclusion

In this paper, we have built a theoretical framework based on Resource-Based View Theory (RBVT), Decision Theory (DT), and Theory of Planned Behavior (TPB) that explains the mediating effects of sustainability and competitive advantage on the relationship between internet banking and BID, and established the hypotheses by evaluating data collected from distinct banks in Congo. The findings show that an element of innovation, namely internet banking, is significantly linked with sustainability and competitive advantage that contribute substantially to bank investment decisions. Both sustainability and competitive advantage partially mitigate the effect of internet banking on banks’ investment decisions (BID). The resource-based view theory is regarded as one of the most important theories in financial literature. The current study aims to apply the concept of resource-based view theory to the financial management and investment decisions of firms. Only a few authors have examined resource-based view theory and decision theory to firms’ financial management and investment decisions in the literature. Another point to note is that previous research has primarily focused on the role of innovative activities as investment predictors. According to the resource-based view theory, financial innovations or techniques influence investment decisions through performance, sustainability, competitive advantage, and stakeholder satisfaction. The current study looked at the role of bank sustainability and competitive advantage, which had previously been overlooked by academics.

The majority of innovation research is focused on the individual or contextual factors that contribute to its improvement, as few businesses can remain viable, prosper, and perform effectively in the competitive real world without innovations and technologies (Anderson et al., 2014). Whereas a limited number of studies are beginning to appear that examine the effects of innovation and investment decisions (Shahwan, 2018; Carboni and Medda, 2019). Our findings broaden innovation theory by analyzing the outputs that involve both its costs and benefits. The outcomes often explain in which situations innovators frequently get benefits, and at other times, the price they pay for spreading creative ideas. This study establishes a framework for future research that will assist businesses in determining how and when to stimulate the positive effects of their employees’ creative activities while minimizing adverse impacts.

This research has important implications for organizational managers, and it adds to the body of knowledge by expanding on previous research. As twenty first-century businesses are operating in an unexpected and difficult business environment, the rate of growth is anticipated to accelerate. Therefore, the employees’ capabilities will grow because of gaining more knowledge and exchanging it with others. It reinforces a sense of commitment to the assigned tasks. The present investigation makes an important contribution to the Resource-Based View Theory and Theory of Planned Behavior (TPB). The usage of innovation to evaluate the investment decision is directly linked to how Resource-Based View Theory and TPB are comprehended. Investment decisions are affected by the Fintech services, sustainability, competitive advantage, and mutual coordination between managers, employees, and customers inside the company. This enables the consideration of both customers and employees in the decision-making process for the survival of the corporation. The effects of BS and BCA on BID were included in the study model. These findings demonstrate that the nexus between IB and BID can be bettered by enhancing both mediators. To this end, this research adds the contingence viewpoint to the managerial investigation.

Apart from theoretical implications, this research offers clear guidance to a number of banking practitioners, including human resource practitioners, team leaders, decision-making managers, and management information systems managers. Increased investment and promotion of effective innovation tend to increase competitive advantage and long-term viability. HR professionals concentrate on enhancing the company’s image. Fintech is an effectual practice that assists organizations to gain a competitive advantage and strengthen their sustainability, which ultimately improves investment decisions. Proper implementation of innovative technology leads to BID. Moreover, the outcomes of this paper show that banks’ managers should concentrate on innovation as well as other factors that increase performance, competitive advantage, and long-term viability, all of which would benefit BID as a whole. This research also aids the researchers in clarifying the importance of IB, BS, and BCA in strengthening the BID. In their studies, the researchers can also use this empirical evidence framework to see if there are any other contributory factors that fit into this model.

## Data Availability Statement

The raw data supporting the conclusions of this article will be made available by the authors, without undue reservation.

## Author Contributions

JP-A: conceptualization, methodology, software, visualization, investigation, writing—reviewing and editing, data curation, and writing—original draft preparation. MW: conceptualization, supervision, validation, investigation, and reviewing and editing. Both authors read and approved the final manuscript.

## Conflict of Interest

The authors declare that the research was conducted in the absence of any commercial or financial relationships that could be construed as a potential conflict of interest.

## Publisher’s Note

All claims expressed in this article are solely those of the authors and do not necessarily represent those of their affiliated organizations, or those of the publisher, the editors and the reviewers. Any product that may be evaluated in this article, or claim that may be made by its manufacturer, is not guaranteed or endorsed by the publisher.

## Appendix

**TABLE A1:** Study questionnaire.

Items	References
**“Internet banking (IB)”**	Wu et al., 2012; Rahi et al., 2019
**“IB1.** You feel confident while using e-banking method to access money”	
**“IB2.** Internet banking enables me to complete a transaction quickly”	
**“IB3.** Online banking enhances your effectiveness in doing banking transactions”	
**“IB4.** You find online banking useful”	
**“IB5.** Online banking saves your time”	
**“IB6.** Overall, online banking is easy to use”	
**“Bank sustainability (BS)”**	Njoroge et al., 2019
**“BS1.** Sustainability increases credibility, improves brand value and gain in reputation”	
**“BS2.** Bank sustainability improves Health and safety for workers, and better quality of environment.”	
**“BS3.** Sustainability develops new products and services, increases value to stakeholders and develops new business trends.”	
**“BS4.** Bank sustainability lowers risk, reduces cost, improves access to international financing and community relations, and attracts new customers.”	
**“Bank competitive advantage (BCA)”**	Anwar, 2018
**“BCA1.** Improves product quality and Raise service quality by investing in innovation and technology”	
**“BCA2.** Widening the current network of your bank’s branches”	
**“BCA3.** Increases financing for activities targeting expenditure in information and communication technology”	
**“BCA4.** Making the bank’s products different from those of others”	
**“Bank investment decision (BID)”**	Ogunlusi and Obademi, 2019
**“BID1.** My investment reports better results than expected”	
**“BID2.** My investment in IT and Innovation has demonstrated increased cash flow growth in past 5 years”	
**“BID3.** My investment in Technology innovation has a lower risk compared to the market financial products in general”	
**“BID4.** My investment in sustainability activities has a high degree of safety”	
**“BID5.** My investment proceeds will be used in a way that benefit society”	
**“BID6.** Investment decisions are made independently of how to finance them and increase the firm’s involvement in solving community problems.”	
